# Age-Related Changes in the Retinal Pigment Epithelium (RPE)

**DOI:** 10.1371/journal.pone.0038673

**Published:** 2012-06-11

**Authors:** Xiaorong Gu, Nikolas J. Neric, John S. Crabb, John W. Crabb, Sanjoy K. Bhattacharya, Mary E. Rayborn, Joe G. Hollyfield, Vera L. Bonilha

**Affiliations:** 1 The Cole Eye Institute, Department of Ophthalmic Research, Cleveland Clinic, Cleveland, Ohio, United States of America; 2 Bascom Palmer Eye Institute, University of Miami, Miami, Florida, United States of America; University of Florida, United States of America

## Abstract

**Background:**

Age-related changes in the retina are often accompanied by visual impairment but their mechanistic details remain poorly understood.

**Methodology:**

Proteomic studies were pursued toward a better molecular understanding of retinal pigment epithelium (RPE) aging mechanisms. RPE cells were isolated from young adults (3–4 month-old) and old (24–25 month-old) F344BN rats, and separated into subcellular fractions containing apical microvilli (MV) and RPE cell bodies (CB) lacking their apical microvilli. Proteins were extracted in detergent, separated by SDS-PAGE, digested in situ with trypsin and analyzed by LC MS/MS. Select proteins detected in young and old rat RPE were further studied using immunofluorescence and Western blot analysis.

**Principal Findings:**

A total of 356 proteins were identified in RPE MV from young and 378 in RPE MV from old rats, 48% of which were common to each age group. A total of 897 proteins were identified in RPE CB from young rats and 675 in old CB, 56% of which were common to each age group. Several of the identified proteins, including proteins involved in response to oxidative stress, displayed both quantitative and qualitative changes in overall abundance during RPE aging. Numerous proteins were identified for the first time in the RPE. One such protein, collectrin, was localized to the apical membrane of apical brush border of proximal tubules where it likely regulates several amino acid transporters. Elsewhere, collectrin is involved in pancreatic β cell proliferation and insulin secretion. In the RPE, collectrin expression was significantly modulated during RPE aging. Another age-regulated, newly described protein was DJ-1, a protein extensively studied in brain where oxidative stress-related functions have been described.

**Conclusions/Significance:**

The data presented here reveals specific changes in the RPE during aging, providing the first protein database of RPE aging, which will facilitate future studies of age-related retinal diseases.

## Introduction

The retinal pigment epithelium (RPE) is a cuboidal epithelium containing very long sheet-like apical microvilli that project into a complex matrix, referred to as the interphotoreceptor matrix. At this interface the microvilli interact with the tips of cylindrical photoreceptor outer segments extending from the outer retinal surface. The RPE basal surface is highly infolded and interacts with the underlying Bruch’s membrane [1], [2], an acellular multilayered extracellular lamina separating the RPE from the choriocapillaris.

**Figure 1:**
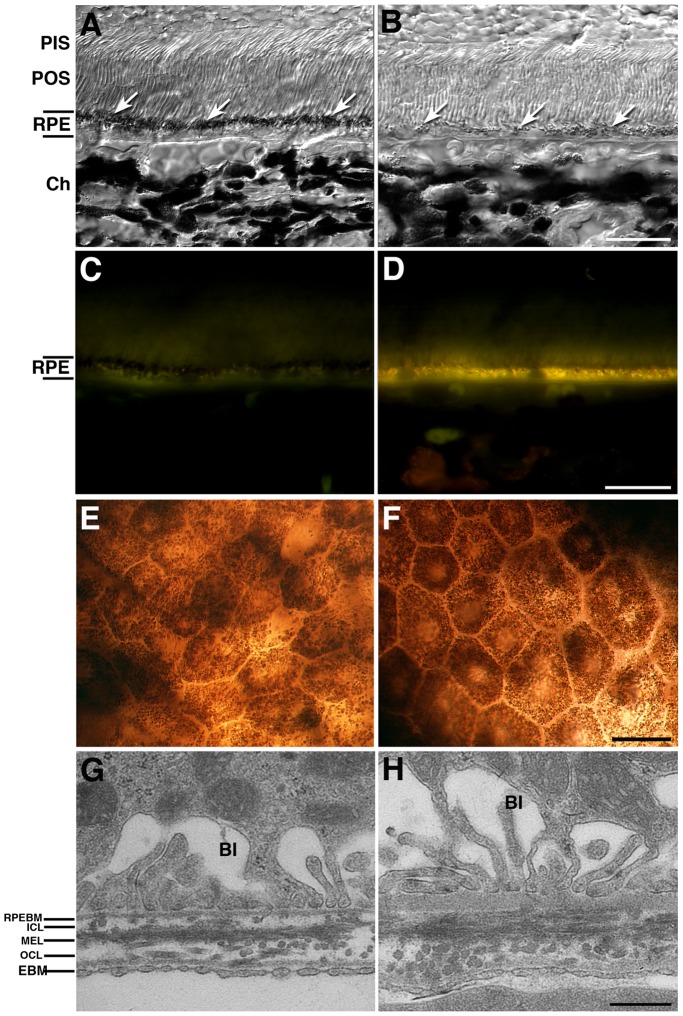
Age-related changes in F344BN rat RPE. Observation of young (3–4 month-old, A, C, E, G) and old (24–25 month-old, B, D, F. H) F1 F344BN hybrid rats reveals several of the RPE age-related changes previously described in humans. Old RPE displays loss of melanin granules (arrowheads in A, B) and accumulation of the age pigment lipofuscin (D), as observed by the presence of increased autofluorescent granules when observed on epifluorescence in the green channel (FITC filter: excitation 495 nm/emission 519 nm) when compared to young RPE (C). Bright-field micrographs of RPE whole-mounts suggested that young RPE cells (E) are frequently smaller than the old RPE cells (F). Additional observation of the old RPE displayed thickening of Bruch’s membrane (BM) and basal infoldings disorganization (H) when compared to young BM (G) processed and analyzed by transmission electron microscopy (TEM). Differential interference contrast microscopy images (DIC) (A, B). BI =  basal infoldings; RPEBM =  RPE basement membrane; ICL =  inner collagenous layer; MEL =  middle elastic layer; OCL =  outer collagenous layer; EBM =  choroidal endothelial cell basement membrane; Bars (A to D) = 20 µm, (E, F) = 200 µm, (G, H) = 0.5 µm.

The RPE performs highly specialized, functions essential for retinal homeostasis. These include phagocytosis of photoreceptor shed outer segments, directional transport of nutrients into and removal of waste products from photoreceptor cells, optimization of ion concentrations in the surrounding tissues, elimination of fluid from the subretinal space, and visual pigment regeneration and transport. The apical microvilli of the RPE play a key role in mediating these activities [3], [4]. On the basolateral side, the proteins present in the RPE basal surface regulate the exchange of nutrients and signaling molecules between the RPE and the choroidal endothelial cells and establish the outer portion of the blood–retina barrier [5].

During aging the RPE undergoes a number of well characterized structural changes, including loss of melanin granules, increase in the density of residual bodies, accumulation of lipofuscin, accumulation of basal deposits on or within Bruch’s membrane, formation of drusen (between the basal lamina of the RPE and the inner collagenous layer of Bruch’s membrane), thickening of Bruch’s membrane, microvilli atrophy and disorganization of the basal infoldings. Although these changes are well known, they progress slowly with time and vary in severity in different individuals. The molecular mechanisms involved in these changes are not completely understood.

Many of the variable factors complicating the analysis of human derived specimens can be eliminated using animal models, which are genetically identical. Animals are housed under identical conditions, restricting environmental effects, and can be examined at identical times/disease points [6]. Therefore, we utilized the F1 hybrid between Fischer 344 and Brown Norway rats (F344BN) in our study.

Previous studies have detailed the ultrastructural descriptions of the degenerating photoreceptor cell nuclei, inner and outer segments, the reactive Muller cells, the breakdown of the outer limiting membrane, and lipofuscin accumulation in the RPE of old Fisher 344 rats. Moreover, the eyes of 24-month-old F344 rats showed progressive changes in the RPE/Bruch’s/choriocapillaris complex that included both diffuse and nodular thickening of Bruch’s membrane as well as vacuole accumulation and collagen deposition. In addition, proliferation of basement membrane and accumulations of broken down organelles, mostly mitochondria, which had ultrastructural similarities to the components of drusen have also been observed [7]–[9].

Proteomics provides a global, unbiased approach for examining changes in protein expression and thus offers the opportunity for discovery of novel signaling events and targets in biology. In this study, we used mass spectrometry to identify proteomic changes with age in rat RPE microvilli (MV) and RPE cell body (CB) fractions. Select proteins were then qualitatively and quantitatively characterized using Western blot and immunocytochemical analysis. Knowledge of the protein changes in the aging RPE cell will provide insight into biochemical processes that support and maintain vision through aging.

**Figure 2:**
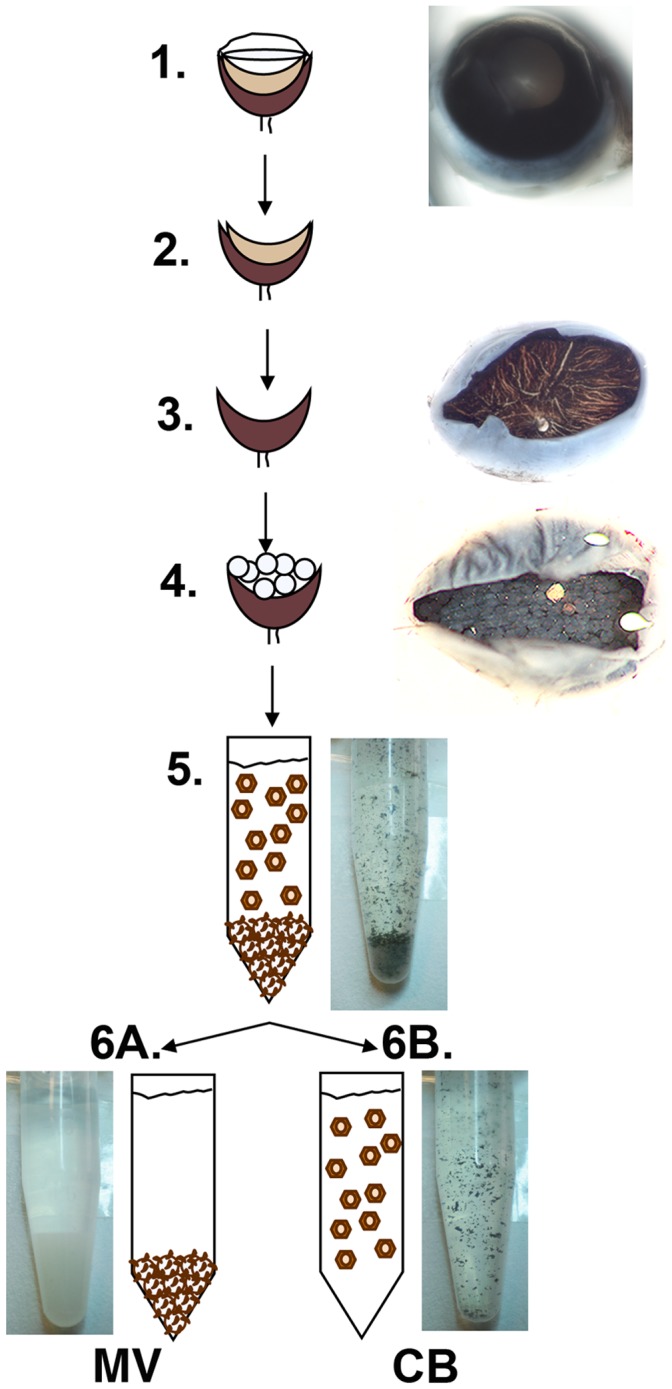
RPE fractions isolation scheme. 1-Mouse eyes are enucleated, 2-the anterior segments are surgically removed, and 3-the retina is removed after enzymatic treatment. 4- WGA-agarose beads are subsequently overlaid on the exposed RPE eyecups. After incubation, 5- the WGA-beads and RPE CB are gently scraped from the eyecups and collected into tubes. 6A- While WGA-agarose macrobeads are allowed to decant to the bottom of the tubes, 6B- the supernatant, with CB, is transferred to a fresh tube, and each fraction is washed and further processed for either biochemistry or morphology assays.

**Figure 3:**
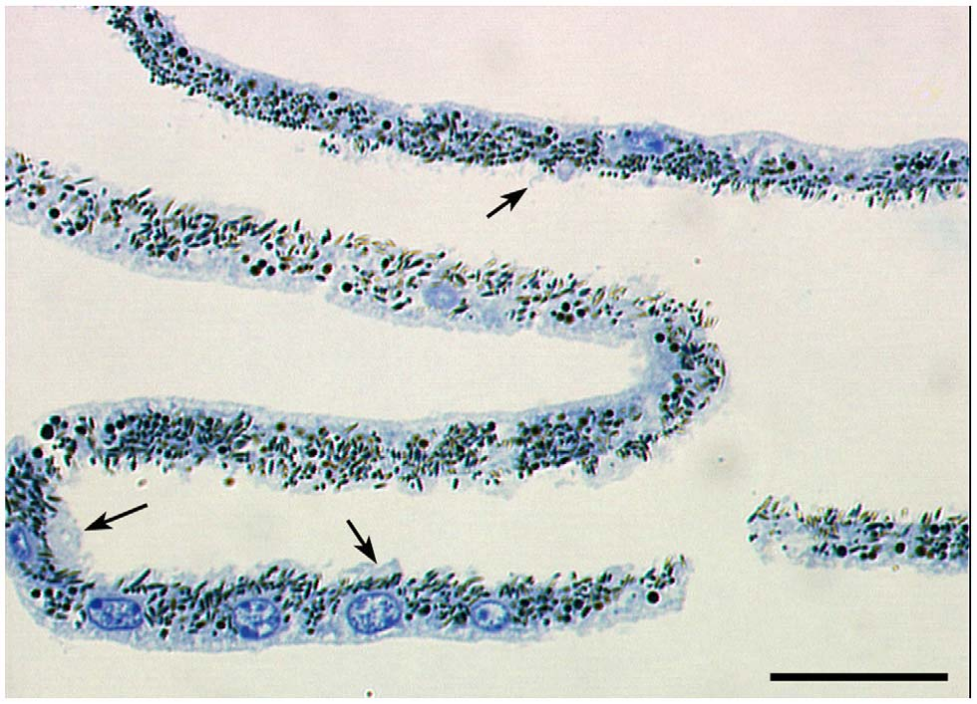
Morphological characterization of cell body (CB)-enriched fraction. Isolated CB cells display normal RPE morphology, without any significant damage to the cell body and monolayer. Most of the RPE cells had their MV removed by the WGA-beads but a few RPE cells (arrows) still have MV on their apical surface. Bar  = 200 µm.

## Results

### The F344BN Model for RPE Aging

The F_1_ F344/BN hybrid model was chosen for the present study because their eyes are pigmented and ensure the purity of the RPE preparation. In addition, the young (3–4 month-old) RPE cells in this model display the typical accumulation of melanin granules on the apical surface (Fig. 1A), lack of lipofuscin accumulation in the cytoplasm (Fig. 1C), presence of small, dense RPE cells (Fig. 1E) and the presence of a well-structured BM (Fig. 1G). On the other hand, the old (24–25 month-old) F344BN rats display several of the RPE age-related changes previously described in humans. Specifically, old RPE displays loss of melanin granules (Fig. 1B), lipofuscin accumulation (Fig. 1D), decreased RPE density with the presence of bigger cells (Fig. 1F), and BM thickening (Fig. 1H).

**Figure 4:**
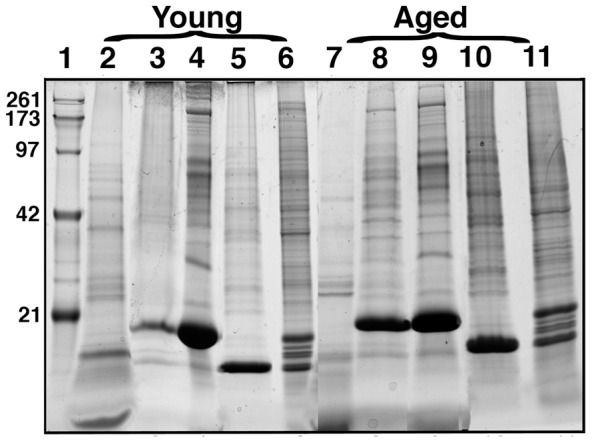
Biochemical characterization of RPE fractions of both young and old rats. Representative one-dimensional coomassie-blue SDS-PAGE of fractions (∼10 µg protein per lane) from young (*lanes* 2 to 6) and old (*lanes* 7 to 11). *Lane 1*, molecular weight markers are indicated in kDa.; *lanes 2 and 7*, detergent-soluble MV fractions; *lanes 3 and 8*, detergent-insoluble MV fractions; *lanes 4 and 9*, detergent-soluble CB fractions; *lanes 5 and 10*, detergent-insoluble CB fractions; *lanes 6 and 11*, rat RPE whole cell lysate.

### Isolation of RPE CB and MV Fractions

To check for alterations in sugar residues exposed by the young adult and old RPE apical surface, eyes were labeled with the WGA lectin, specific to sialic acid and N-acetylglycosamine residues, conjugated to FITC (supplementary Figure S1). In these experiments both whole mount eyecups (supplementary Figure S1A and B) and cryosections (supplementary Figure S1C and D) of eyecups with their RPE apical surface exposed were analyzed. Our observations demonstrated WGA association with the apical surface of both the young adult (supplementary Figure S1A and C) and old (supplementary Figure S1B and D) RPE apical surface. However, the intensity of the labeling was significantly lower in the old rats when compared with the young adults. This observation is in accordance with the ultrastructural observation of lower density of apical microvilli in the old rats described above. Based on the histochemical analyses of the samples we expected to have a lower amount of intact isolated microvilli from the old animals. However, the number of animals used per experiment was shown to be appropriate to generate a well-resolved protein profile.

**Figure 5:**
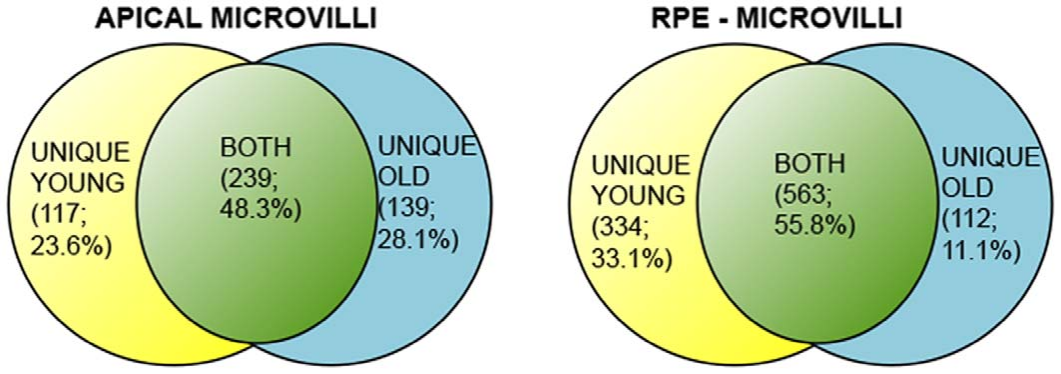
Venn diagram showing overlap of proteins identified in both young and old RPE fractions. Only 11% of the identified proteins were uniquely found in the old RPE CB while 33% of the proteins identified were uniquely found in young CB, supporting significant proteomic changes with age.

**Table 1:** Selected proteins identified on CB fraction of rat RPE.

Proteins	Accession Number^a^	MatchedProtein	ProteinCoverage (%)	Fraction^b^
1-acyl-sn-glycerol-3-phosphate acyltransferase gamma	B0BNL8	18	11.4	Y & A
10 kDa heat shock protein, mitochondrial	P26772	6	43.1	Y & A
2-oxoglutarate dehydrogenase E1 component, mitochondrial	Q5XI78	26	15.2	Y & A
2,4-dienoyl-CoA reductase, mitochondrial	Q64591	27	38.8	Y & A
3-ketoacyl-CoA thiolase A, peroxisomal	P21775	11	26.4	Y & A
40 S ribosomal protein S15a	P62246	10	56.2	Y & A
40 S ribosomal protein S2	P27952	10	22.5	Y
60 S acidic ribosomal protein P2	P02401	5	51.3	Y
Aconitate hydratase, mitochondrial	Q9ER34	95	50.8	Y & A
ADP-ribosylation factor 5	P84083	26	5.6	A
ADP/ATP translocase 1	Q05962	138	53.4	Y & A
AP-2 complex subunit beta-1	P62944	10	12.0	Y
Apoptosis-inducing factor 1, mitochondrial	Q9JM53	16	25.7	Y
Bardet-Biedl syndrome 2 protein homolog	Q99MH9	13	11.15	Y & A
Calnexin	P35565	46	23.7	Y & A
Cathepsin D	P24268	177	39.6	A &Y
Dipeptidyl-peptidase 1	P80067	8	20.8	Y & A
Dipeptidyl-peptidase 2	***Q9EPB1***	8	10.0	***Y***
ELMO domain-containing protein 2	***D3ZNV6***	6	13.3	***Y & A***
Ezrin (p81) (Cytovillin) (Villin-2)	P31977	69	35.8	Y & A
Glutathione peroxidase 1	P04041	14	57.2	Y
Heat shock 70 kDa protein 1A	Q07439	7	5.9	Y
Heat shock protein HSP 90-alpha	P82995	44	12.8	Y & A
Histone H4	P62804	47	83.5	Y & A
Integrin beta-1	P49134	15	19.3	Y & A
Leucine zipper-EF-hand-containing transmembrane protein 1, mitochondrial	Q5XIN6	14	22.5	Y & A
Lysosome-associated membrane glycoprotein 2	P17046	23	11.9	Y & A
Malate dehydrogenase, mitochondrial	P04636	55	64.8	Y & A
Monocarboxylate transporter 3	O70461	14	15.04	Y & A
Myomegalin	Q9WUJ3	15	5.3	A
NG,NG-dimethylarginine dimethylaminohydrolase 1 (Dimethylargininase-1)	O08557	11	27.7	Y
Oxysterol-binding protein-related protein 6	Q8BXR9	19	11.5	Y & A
PDZ domain-containing RING finger protein 3	P68907	31	10.5	Y
Peroxiredoxin-6	O35244	12	55.4	Y & A
Phospholipid hydroperoxide glutathione peroxidase, nuclear	Q91XR8	6	12.3	Y
Protein DJ-1	O88767	11	55.6	Y & A
Protein ERGIC-53	Q62902	7	9.3	Y & A
Ras-related protein Rab-8A	P35280	14	14.0	Y & A
Retinal pigment epithelium-specific 65 kDa protein	O70276	260	49.2	Y & A
Rootletin (Ciliary rootlet coiled-coil protein)	Q8CJ40	58	6.1	Y & A
Solute carrier family 2, facilitated glucose transporter member 1 (Glut-1)	P11167	55	13.0	Y & A
Sulfated glycoprotein 1 (SGP-1) (Prosaposin)	P10960	40	25.5	Y & A
Transmembrane emp24 domain-containing protein 4	B5DEM3	30	12.8	A
Ubiquitin carboxyl-terminal hydrolase 19	Q6J1Y9	82	6.6	Y & A
Zinc transporter ZIP4	A0JPN2	9	6.7	Y

a Swiss Protein database accession numbers are shown; for links use the EXPASY server at http://expasy.org/sprot/.

b Fractions: Y  =  young, A  =  aged and Y & A  =  young and aged fractions.

A schematic overview of the CB and MV isolation method is presented in Fig. 2. The procedure relies on the interaction of N-acetylglucosamine and sialic acid-containing glycoconjugates present in abundance on the free surface of epithelial microvilli [10]–[11] with the WGA lectin conjugated to agarose macrobeads. The eyes of young and old rats were enucleated and the anterior segments and vitreous removed. Eyecups were incubated in hyaluronidase then the neural retina was peeled away mechanically, exposing the RPE. WGA-agarose macrobeads suspended in buffer were added into each eyecup and incubated for 3–4 hrs at 4°C. The WGA-macrobeads were scraped off the eyecups using a syringe with 23 gauge needle. At the same time, further scraping of the eyecups leads to the detachment of cell body sheets. This procedure allowed the collection in an eppendorf tube of both RPE CB (on the supernatant), and MV attached to the WGA-agarose macrobeads pelleted at the bottom of the tube. WGA-agarose macrobeads were allowed to decant to the bottom of the tube and the supernatant (with the cell bodies) was transferred to a new tube. Both fractions were extensively washed with buffer and further analyzed. Subsequent analysis of the CB fraction showed normal RPE morphology, without any significant damage to the cell body and monolayer (Fig. 3), indicating that this procedure allows for selective isolation of intact RPE monolayers mostly deprived of microvilli and free of contamination with choroidal tissue.

**Figure 6:**
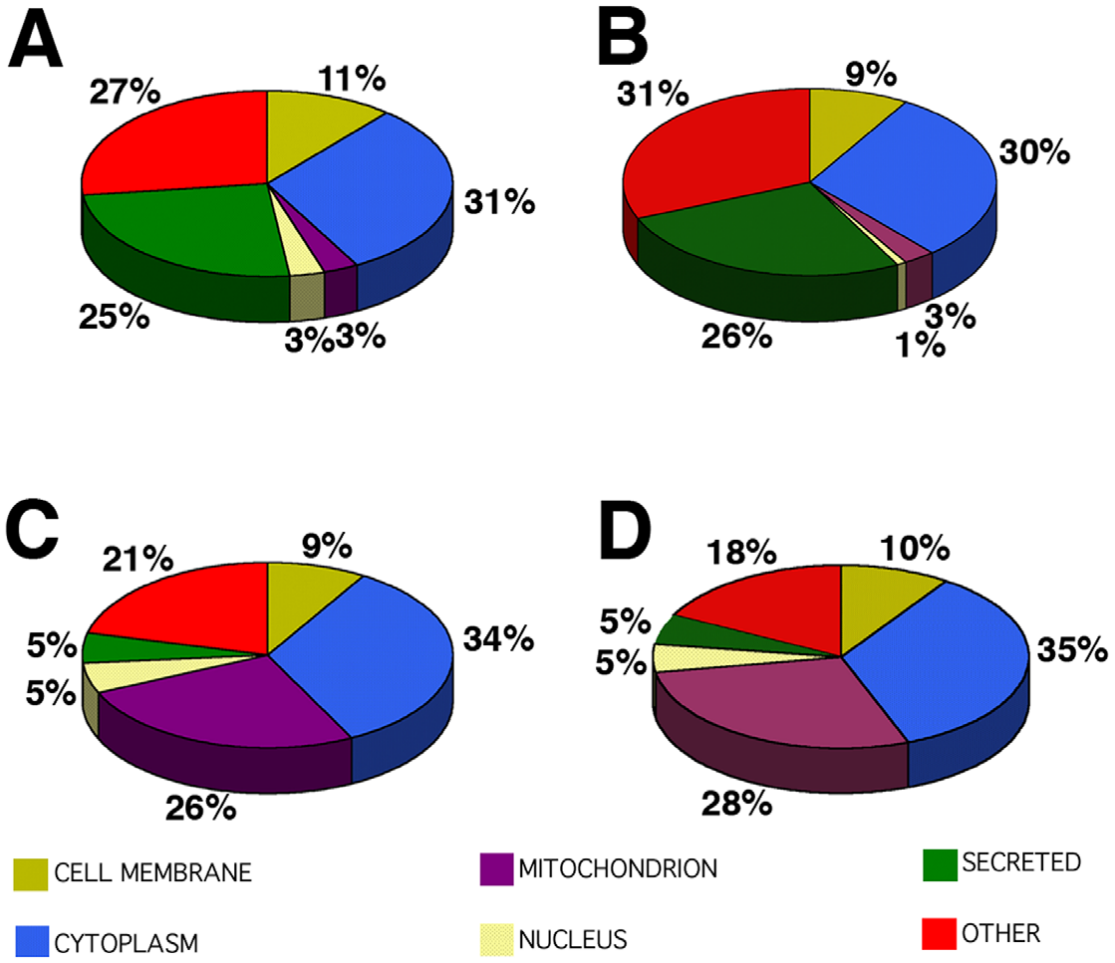
Classification of differentially expressed proteins in RPE fractions according to their cellular localization. Overall profile of cellular localization of the proteins identified from both young and old MV (A and B) and CB (C and D). The pie charts display the classification of the differentially expressed proteins listed in supplementary Tables S1, S2, S3, S4 into 6 distinct cellular compartments. The distribution frequencies in regard to the specified categories within the given chart pie are indicated in percentage of the total number of entries.

The enriched fractions were further fractionated by extraction with a triton X100-containing buffer to decrease the protein complexity of each fraction. When the RPE fractions were resolved on a gradient SDS-PAGE, they showed distinctly different banding pattern compared to total RPE lysates (Fig. 4). The young MV soluble fraction showed several prominent protein bands (ca. 100, 90, 80, 60, 50, 40 and 30–25 kDa) that were not as prominent in the young MV insoluble fraction (compare Fig. 4, lanes 2 and 3). In contrast, several major bands in the molecular weight range 280–150 and 100–40 kDa were prominent in the young soluble CB fraction (Fig. 4, lane 4). A remarkable difference in the protein content of the old MV soluble fraction was noticed, with the presence of prominent protein bands with 50, 40 and 35–25 kDa (Fig. 4, lane 7). The old MV insoluble fraction included several major bands in the molecular range 180–10 kDa. In addition, the old CB soluble fraction included prominent protein bands with 300 and several major bands in the molecular range 180-10 kDa (Fig. 4, lane 9). On the other hand, the old CB insoluble fractions were very similar to the young CB insoluble fraction (compare Fig. 4, lanes 5 and 10).

**Figure 7:**
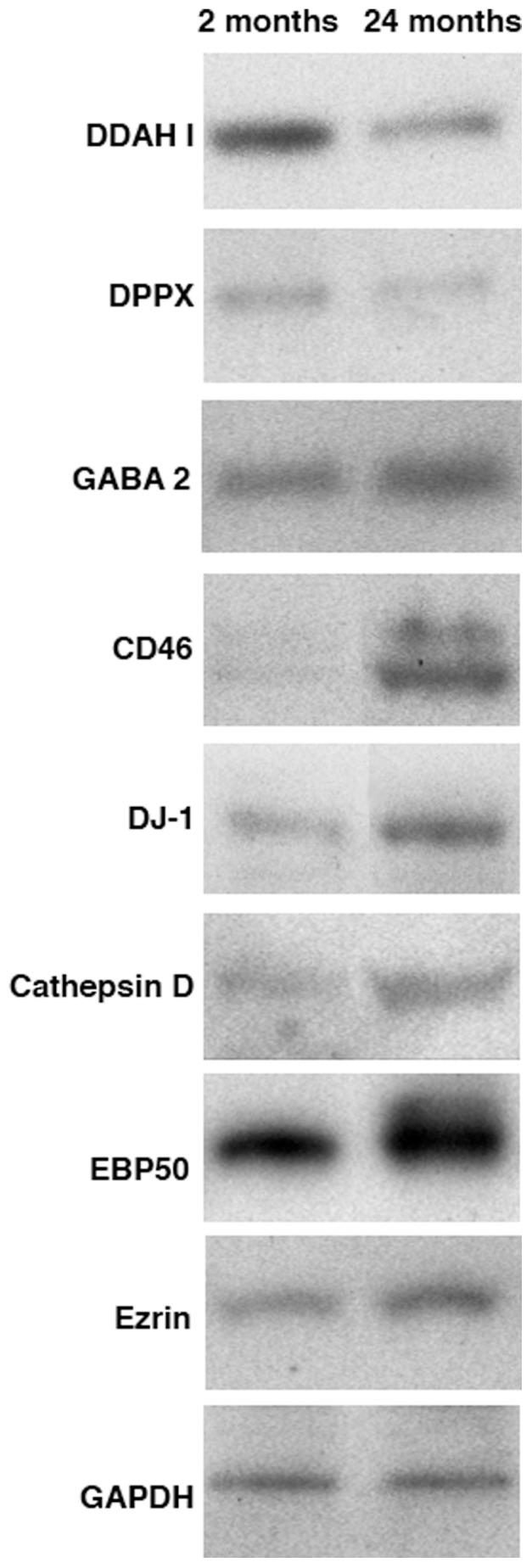
Levels of identified proteins in RPE lysates. Both young (2 months) and old (24 months) RPE cells were harvested, lysed. 20 µg of protein of each sample was separated on a 4–20% SDS gel, transferred to PVDF membranes and probed with antibodies specified followed by ECF detection of immunoreactivity. Decreased content of DDAH I and DPPX were observed in old RPE lysates. Contrary, increased content of GABA2, CD46, DJ-1, cathepsin D, EBP50 and ezrin were detected in old RPE lysates.

**Table 2:** Age- elated changes in the levels of expression of selected RPE protein.

Age	DDAH I	DPPX	GABA 2	CD46	DJ-1	Cath. D	EBP50	Ezrin
2 m.o	260.78±25.08	317.12±33.04	106.55±30.56	104.86±14.94	103.81±11.86	141.58±16.89	46.19±12.78	103.81±11.86
24 m.o.	136.05±4.88	125.56±54.80	229.58±23.45	186.59±23.79	436.44±61.72	266.26±30.61	237.41±34.02	436.44±61.72

Values correspond to mean relative intensity adjusted to GADH relative intensity ± SE. For each protein depicted, three separate experiments were performed with the young RPE lysates and four separate experiments were performed with the old RPE lysates. For each experiment, RPE cells from 8–10 eyes were pooled. Bands were quantified using ImageJ. Results from three independent experiments were combined to calculate mean relative intensity.

### Identification of RPE Proteins

LC MS/MS sequence analyses of peptides from SDS-PAGE gel bands following in situ trypsin digestion resulted in the identification of 356 proteins in young MV and 378 proteins in old MV, 897 proteins in young CB, and 675 proteins in the old CB (Supplementary Tables S1, S2, S3, S4). Our analysis has revealed that about 48% of all identified proteins were detected at both ages in F344BN RPE apical MV and 56% in the CB fractions (Figure 5). Interestingly, about 30% fewer proteins were identified in the old relative to the young CB fraction. We previously characterized the microvilli fraction isolated using the described procedure [13]. A summary of selected proteins identified in both the young and old CB fraction is shown in Table 1. The RPE CB fraction was enriched in nuclei, mitochondrial, peroxisomal, and lysosomal proteins among others. These results therefore provide an unbiased account of proteins present in the RPE CB.

**Figure 8:**
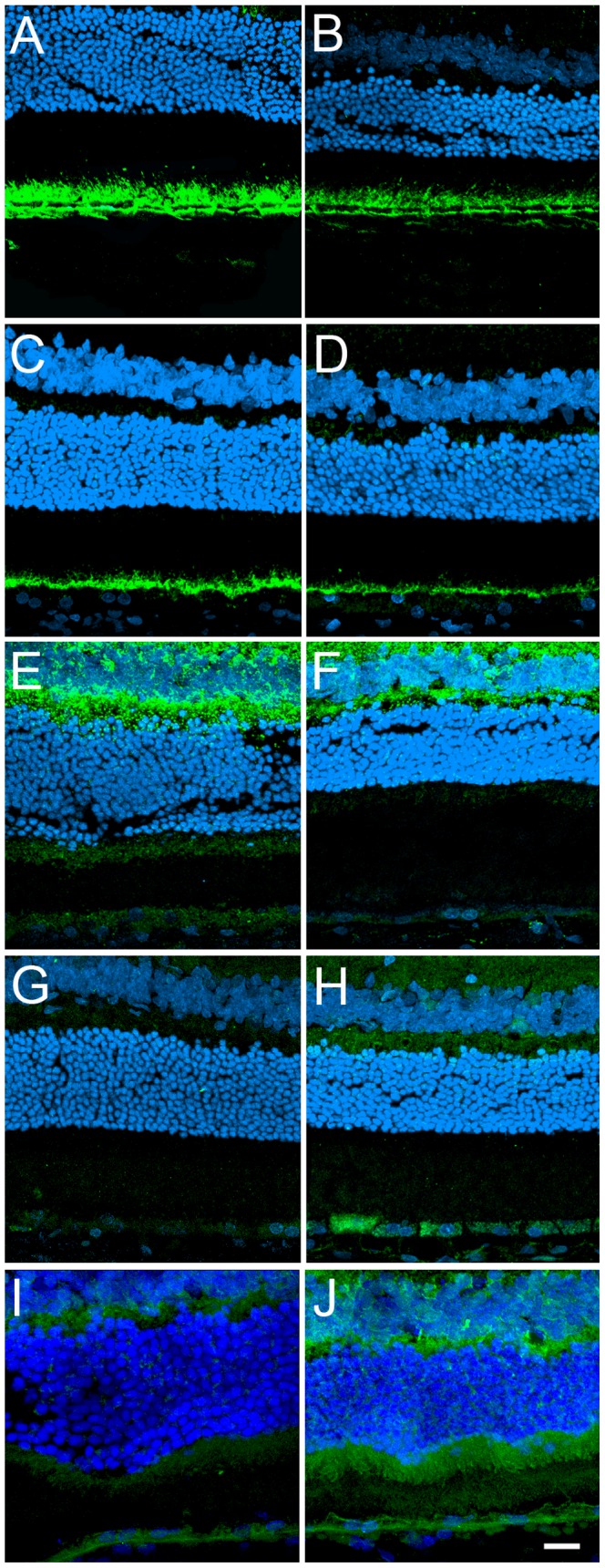
Tissue localization of identified proteins in RPE. Both young (A, C, E, G, I) and old (B, D, F, H, J) F344BN eyes were fixed and immunostained for Glut-1 (A, B), MCT1 (C, D), SOD2 (E, F), BLV (G, H) and DJ-1 (I, J). Cell nuclei were labeled with TO-PRO-3. While Glut-1 (A, B) is localized to both the apical MV and basal surface of the RPE, MCT1 (C, D) is only present in the MV. On the other hand, SOD2 (E, F) and BLV (G, H) are mostly cytoplasmic. The protein DJ-1 (I, J) is present both in the plasma membrane and cytoplasm of the cells. Bar  = 20 µm.

**Figure 9:**
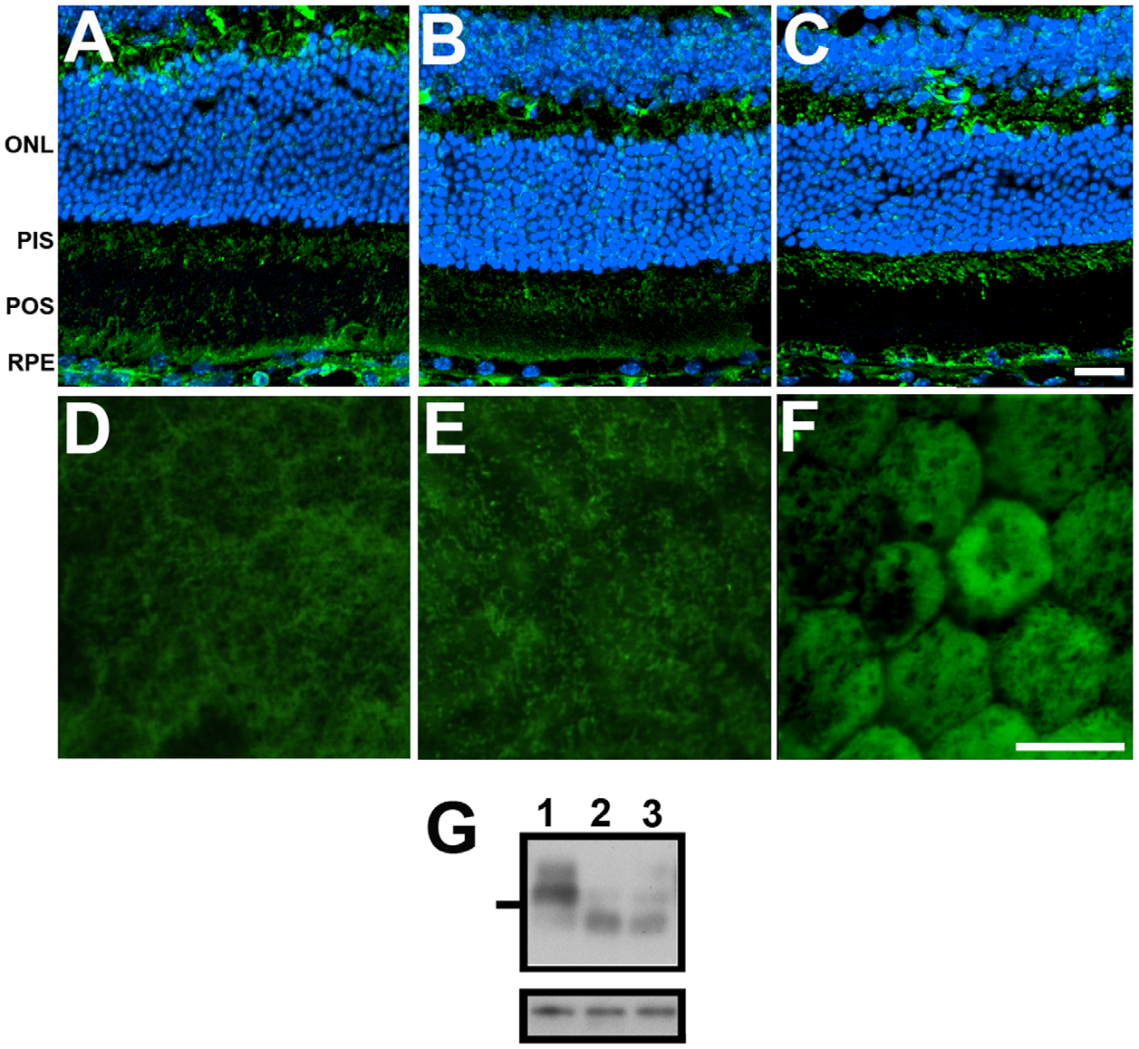
Age-related expression of collectrin. Immunohistochemical analyses. The retina of 3 (A) and 6 month-old (B) and 1 year-old (C) C57Bl mice probed with anti-collectrin antibody. Cell nuclei were labeled with TO-PRO-3. Collectrin is localized to both the apical MV and basal surface of the RPE. Apical localization of collectrin was confirmed when 3 (D) and 6 month-old (E) and 1 year-old (F) mice eyecups with the RPE exposed were labeled with collectrin antibody. G. Representative Western of 20 µg protein from 3 (lane 1) and 6 month-old (lane 2) and 1 year-old (lane 3) RPE lysates reacted with collectrin (upper panel) and actin antibodies (lower panel).

PANTHER analysis was used to evaluate gene ontology and protein function and cellular localization (Fig. 6, A to D). Concerning cellular distribution, proteins were localized in the mitochondrion, cytoplasm, cell membrane, nucleus, secreted or other localization. Both the apical MV (Fig. 6, A and B) and CB (Fig. 6, C and D) fractions were very similar in their composition, independent of the age. Membrane and cytoplasmic protein composition of the apical microvilli (31 and 30%) and CB (34% and 35%) fractions was similar in both ages. However, apical MV displayed a high composition of secreted proteins (25 and 26%), while CB fractions displayed high composition of mitochondrion protein (26 and 28%) in both ages analyzed.

### Validation of Identified Proteins by Immunoblot and Immunohistochemistry Analysis

To further characterize age-related changes in RPE, we used Western blot to probe select identified proteins in total RPE lysates (Fig. 7). In RPE lysates from older animals we detected higher levels of the membrane cofactor protein CD46 and the sodium- and chloride-dependent GABA transporter 2 (GABA 2), of the protein DJ-1, of ERM-binding phosphoprotein 50 (EBP50) and ezrin, and the lysosomal protein cathepsin D. In contrast, we detected lower levels of NG,NG-dimethylarginine dimethylaminohydrolase 1 (DDAH I) and dipeptidyl peptidase X (DPPX) in old RPE (see Fig. 7 for representative immunoblots and Table 2 for quantification).

Using confocal microscopy, qualitative comparisons were pursued between the young and old RPE. Immunolocalization of plasma membrane proteins such as Glut-1 (Fig. 8A and 8B), and monocarboxylate transporter 1 (MCT1) (Fig. 8C and 8D) were observed in the RPE. In addition, proteins involved in oxidative stress response such as superoxide dismutase [Mn] (SOD2) (Fig. 8E and 8F) and biliverdin reductase (BVR) (Fig. 8G and 8H) were also localized in young and aging RPE. The distribution of DJ-1, a protein involved in oxidative stress response and identified here for the first time in RPE, was also analyzed (Fig. 8I and 8J). Results suggest a significant decrease in the immunolocalization of Glut-1 (Fig. 8B), MCT1 (Fig. 8D), and SOD2 (Fig. 8F) in the old RPE. However, an increase in the levels of expression of BVR (Fig. 8H) and DJ-1 (Fig. 8J) was suggested by immunofluorescence of cryosections of old RPE.

### Differential Content of Collectrin in Aging RPE

Collectrin was detected for the first time in RPE and its abundance found to change with age. To validate the presence of collectrin in the RPE, tissue from young and old mice were reacted with antibodies specific to collectrin (Fig. 9). Cryosections of both young and old mice retinas were used for immunolabeling because the commercially available antibody does not recognize rat collectrin (Fig. 9A to C). Collectrin was present both in the apical and basal surfaces of RPE from 3month-old (Fig. 9A), 6 month-old (Fig. 9B) and 1 year-old (Fig. 9C) mice. Localization of collectrin to the RPE apical microvilli was confirmed when mice eyecups with the RPE exposed were labeled with an antibody to collectrin. As previously observed, RPE apical microvilli from 3 and 6 month-old and 1 year-old mice were labeled with collectrin antibody (Fig. 9D to 9F). Finally, reaction of 3 month-old RPE whole cell lysates with collectrin antibody revealed the presence of a unique band with molecular weight ∼30 kDa corresponding to collectrin (Fig. 9G, lane 1). However, RPE lysates from mice 6 month-old (Fig. 9G, lane 2) and 1 year-old (Fig. 9G, lane 3) displayed a decreased molecular weight.

## Discussion

Previously, we have described a simple and efficient method to isolate intact RPE apical MV [12]–[13]. In the present study, we report new methodology that allows for the additional isolation of the RPE cells that remain following the loss of their apical microvilli (CB). We also carried out a detailed mass spectrometry analysis of both RPE fractions in young and old rats. The MV fraction was previously characterized [12]–[13]. Morphological observation of the CB fraction showed normal RPE morphology, without any significant damage to the cell body and monolayer. Moreover, this fraction was free of contamination from choroidal cells. In addition, we were able to show by proteomic analysis that this fraction was highly enriched in mitochondrial and nuclear proteins when compared to the MV fraction. In the present study, we utilized this new methodology to isolate different RPE fractions and carried out proteomic analysis of RPE from F344BN rats of various ages to study the aging processes in these cells.

The aging RPE displays a number of key functional, structural and physiological changes, several of which are notable at the ultrastructural level [14]–[16]. These include loss of melanin granules, increase in the number of residual bodies, accumulation of the age pigment lipofuscin, accumulation of basal deposits on or within Bruch’s membrane, formation of drusen (between the basal lamina of the RPE and the inner collagenous layer of Bruch’s membrane), thickening of Bruch’s membrane, RPE microvilli atrophy and disorganization of basal infoldings [17]; [5] [18]–[19]. However, the precise molecular mechanisms for these changes are not well understood. In the present study, we showed that many of these changes are observed in the old F344BN RPE. Our study has identified hundreds of common proteins present in both young and old RPE fractions. These results suggest that RPE aging is regulated in great part by mechanisms such as post-translational modifications and regulation of levels of expression of proteins rather than by the expression of a set of unique proteins in each age.

Previous studies carried out to characterize differences between young adult and old RPE samples have shown through different experimental approaches that proteins involved in metabolism, energy generation, transport, anti-oxidation, translation, and protein folding are specifically affected by aging [20]–[22]. Our Western analysis of proteins present in RPE lysates revealed increased levels of the lysosomal proteins cathepsin D, of the cytoplasmic proteins DJ-1, ezrin and EBP50, and the plasma membrane proteins CD46 and GABA 2. Conversely, we observed decreased levels of the enzymes DDAH I, DPPX. Our results are in agreement with previous studies that described an overall increase in the activities of cathepsin D and acid phosphatase as a function of age [23]. In addition, it was also reported that EBP50 levels were increase in old CD4+ T cells [24]. DDAH II protein expression was decreased significantly in old endothelial cells compared with young cells [25].

Oxidation is a very important mechanism in aging [26]–[28]. The decrease of antioxidant enzymes such as catalase, gluthathione peroxidase 1, and [Cu-Zn] superoxide dismutase, was also detected in the old F344BN retina sections. The observed decrease in SOD labeling in old RPE is in agreement with previously published data that reported decrease in the activity of these enzymes with aging [29].

Our studies identified for the first time the multifunctional protein DJ-1 in the RPE cells. DJ-1 is ubiquitously expressed in many tissues including the brain. Recent studies suggest that DJ-1 might serve as antioxidant, redox-sensitive molecular chaperone and transcription regulator [30]–[33]. We demonstrate here for the first time that DJ-1 is expressed in RPE and photoreceptor cells. Moreover, we have shown that DJ-1 expression increased in the old eyes, which are characterized by the increased presence of reactive oxidative species. Our observations suggest a correlation between DJ-1 levels of expression and oxidative stress. The increased presence of this protein in old RPE seems to be related to the increased presence of reactive oxygen species in the old RPE. Further experiments will address the role of DJ-1 in oxidative stress signaling.

Another protein identified for the first time in the RPE during this study was collectrin. Collectrin was first described as a protein of unknown function localized to cytoplasm and apical membrane of renal collecting ducts [34]. Later, extrarenal expression has been reported in several tissues and cell lines, including pancreatic β-cells, heart, liver, corpus callosum, hippocampus and MIN6 cells, a mouse insulinoma cell line [35]. More recent studies demonstrated that collectrin is localized to the apical brush border of proximal tubules in mouse kidney, where it is associated with several amino acid transporters and is critical for their normal distribution and function [36]–[37]. An important role of collectrin in pancreatic beta cell proliferation and insulin secretion was suggested, based on *in vitro* experiments in transfected cell lines and transgenic mice overexpressing collectrin in beta cells [35]. Many of the described functions of collectrin are relevant to the phenotype of diabetes. Collectrin in RPE is likely to impact RPE amino acids transport that could contribute to the changes observed during RPE aging. We show that collectrin molecular weight changes as the RPE ages. We believe that this change is the consequence of post-translational modifications. Further analysis will investigate the functional significance of the observed changes.

We also investigated how the differently regulated proteins were integrated into specific regulatory and signaling pathways in the different RPE fractions obtained. Biological relevant networks were drawn through the use of Ingenuity Pathway Analysis (IPA) and several major pathways or networks were identified. Networks with the top five scores for each fraction are shown in supplementary Tables S5 and S6. Networks were ranked according to their degree of relevance to the Network Eligible Molecules found in our proteomic dataset populating each network and their degrees of connectivity compared with all the molecules in the Ingenuity Knowledge Base. The top five networks achieved remarkably high *p* scores between 45 and 32, and accounted for 28 to 22 focus molecules in the MV fraction (Suppl. Table S5). On the other hand, the top five networks in the CB fractions displayed *p* scores between 47 and 37, and accounted for 33 to 26 focus molecules (Suppl. Table S6). Analysis showed that small molecule biochemistry, lipid metabolism, molecular transport, neurological disease, and molecular transport were among the top two network pathways detected in the young MV fraction while small molecule biochemistry, free radical scavenging, drug metabolism, hematological system development and function, organismal functions and cancer were among the top two scored networked in the old MV fraction. On the other hand, genetic disorder, hematological disease, organismal injury and abnormalities, protein synthesis, cancer, and cell death were among the top two scored network pathways in the young CB fraction while cancer, cell death, neurological disease, energy production, nucleic acid metabolism, and small molecule biochemistry were among the top scored network pathways detected in the old CB fraction.

In addition, we were also able to identify common network containing proteins involved in lipid metabolism, molecular transport, and small molecule biochemistry pathways in all the fractions analyzed (supplementary Figure S2). The network analysis revealed that protein interactions in lipid metabolism, molecular transport and small molecules biochemistry pathways significantly changed between fractions with aging. The networks present in the MV of young (supplementary Figure S2A) RPE was very complex and involved complex interactions between several proteins.

In summary, our morphological and biochemical data shows a rapid, reliable and reproducible method for generating two RPE fractions, namely MV and CB. Our results provide the first proteomic database on RPE aging. Therefore, the proteins identified in this study may constitute future treatment targets for age-related retinal diseases.

## Methods

### Ethics Statement

All animal work was conducted in compliance with the Animal Welfare Act and Public Health Services policies, and under the oversight and approval of the Cleveland Clinic Institutional Animal Care and Use Committee (IACUC, protocol number ARC 09054).

### Aging Rat Model

The F1 hybrid between Fisher 344 (F344) and Brown Norway (BN) (F344BN) rats used in these studies were purchased from The National Institute on Aging (NIA) old rodent colonies (Harlan Sprague Dawley, Inc., Madison, WI). Animals were kept on a 12 hours light/dark cycle. Our observations were carried out in young adults (3–4 month-old) and old (24–25 month-old) rats. All animals were handled in a humane manner according to the guidelines of an approved protocol from the Cleveland Clinic IACUC.

### Isolation of RPE Apical Microvilli (MV) and RPE Cell Body (CB) Fractions

A modification of previously described procedure [10] was performed to isolate both the MV and CB fractions. In brief, F344BN rats were sacrificed by CO_2_ asphyxiation, and the eyes were enucleated. The anterior segments were removed and the eyecups with the exposed neural retina were incubated in 320 U/ml bovine testes hyaluronidase (Sigma Chemical Co., St. Louis, MO) in Hank’s buffered solution (HBSS, Mediatech, Inc.) for 1 h at 37°C. The neural retina was peeled off from the RPE. Eyecups with the exposed RPE were extensively washed with Tris-buffered saline (TBS) +1 mM CaCl_2_ (TBS+C) for 1 h at 4°C followed by incubation with WGA-agarose macrobeads (Sigma) in TBS for 3–4 hrs at 4°C. WGA-beads were gently scrapped from the eyecups and transferred to 15 ml tubes. WGA-agarose macrobeads with MV were allowed to decant to the bottom of the tube and the supernatant (with the CB) was transferred to a new 15 ml tube. WGA-agarose macrobeads with MV were washed extensively with PBS after decanting, and processed for biochemical analysis. CB fractions were centrifuged 5 min. at 4000 g and pelleted. For triton extraction, WGA agarose macrobeads with MV and CB fractions were incubated in extraction buffer MES (0.5% triton X-100, 50 mM MES, 5 mM MgCl_2_, 3 mM EGTA, pH 6.4) for 40 secs at RT. Detergent-soluble fractions of both MV and CB were precipitated in 85% acetone overnight at −20°C followed by centrifugation at 300 g for 10 min. at 4°C. Detergent-soluble and -insoluble material of both MV and CB were dissolved in 2×SDS sample buffer and boiled for 4 min at 100°C.

### Sample Preparation for Proteomics Analysis

Detergent-soluble and –insoluble fractions were combined, yielding MV and CB preparations used for proteomic analysis. Protein concentration was determined by BCA assay. (Pierce). About 10 µg of protein from each preparation was resolved by SDS-PAGE on 4–20% Novex®-Tris-Glycine gel (Invitrogen Corporation, Carlsbad, CA). The gel was stained with colloidal Coomassie blue (Code Blue, Pierce), and protein bands were cut from top to bottom into ∼2 mm slices. Gel slices were washed, reduced with 10 mM DTT, alkylated with 55 mM iodoacetamide, and digested *in situ* with trypsin. Tryptic peptides were extracted three times with 0.1% formic acid in 50% acetonitrile and water, and dried with Speed Vacuum.

### Protein Identification by on-line LC MS/MS

Dried peptides from each gel pieces were re-suspended in 0.1% formic acid/2% acetonitrile/98% water, and analyzed by liquid chromatography tandem MS (LC MS/MS) with a CapLC XE system (Micromass, Beverly, MA) and a quadrupole time-of-flight mass spectrometer (QTOF2, Micromass) as described (ref x). Peptides were separated on a 75 µm×5.5 cm Biobasic C18 column (New Objective, Cambridge, MA) by using aqueous formic acid/acetonitrile solvents, a flow rate of 250 nl/min, and a gradient of 5–30% acetonitrile over 25 min followed by 80% acetonitrile for 2 min. Protein identification utilized MASSLYNX 4.1 software (Waters), the Mascot search engine (Matrix Science, version 2.1), and the Swiss-Protein sequence database (version 56.0). The Swiss Protein database search parameters included all Rodent entries, 3 missed tryptic cleavage sites allowed, precursor ion mass tolerance  =  0.8 Da, and fragment ion mass tolerance  =  0.8 Da. *S*-carboxyamidomethyl on Cystine was allowed for fixed protein modification and oxidation on Methionine was allowed for variable protein modification. A minimum Mascot ion score  =  25 was used for accepting all peptide MS/MS spectra. Two unique peptides per protein were required for all protein identifications. To estimate false discovery rates, all peptide ms/ms spectra were searched manually (Matrix Science, version 2.2) against a randomized decoy database constructed with a script at www.matrixscience.com/help/decoy_help.html. Excel locally written macros utilizing the Swiss Protein database and previously described have been used to provide information on relative intensity and subcellular localization of the detected peptides. In addition, analysis of possible pathway networks was performed with Ingenuity Pathways Analysis 8.0 (Ingenuity Systems, Inc., Redwood City, CA).

### Bioinformatic Analyses

Functional classification of proteins was performed with “The Protein ANalysis THrough Evolutionary Relationships" (PANTHER) System (available at www.pantherdb.org). For PANTHER analysis, Swiss Protein accession numbers were converted to Reference Sequence protein accessions using the **D**atabase for **A**nnotation, **V**isualization and **I**ntegrated **D**iscovery (available at http://david.abcc.ncifcrf.gov). Analysis of possible pathway networks was performed with Ingenuity Pathways Analysis 8.0 (Ingenuity® Systems).

### Immunocytochemistry of Identified Proteins

To confirm the localization of some of the proteins identified by LC MS/MS analysis, immunohistochemical assays were performed using cryosections of rat eyes. Eyes were enucleated and fixed by immersion in 4% paraformaldehyde made in PBS for 3 h at 4°C, and subsequently the anterior segments were removed. For cryosectioning, eyecups were fixed as described above, quenched with 50 mM NH_4_Cl made in PBS for 1 h at 4°C, infused successively with 15% and 30% sucrose made in the same buffer and with Tissue-Tek “4583" (Miles Inc., Elkhart, IN). For labeling, sections were washed to remove embedding medium, blocked in PBS supplemented with 0.3 mM CaCl_2_+1 mM MgCl_2_+1% BSA (PBS/CM/BSA) for 30 min, and incubated with the specific antibodies in PBS/CM/BSA overnight at 4°C. The sections were washed in PBS/CM/BSA and incubated with secondary antibodies coupled to Alexa 488 (Molecular Probes, Eugene, OR) for 1 h at RT. Cell nuclei were labeled with 1 mM TO-PRO-3 (Molecular Probes) in PBS for 15 min. A series of 1 µm *xy* (*en face*) sections were collected using a laser scanning confocal microscope (Leica TCS-SP, Exton, PA). Each individual *xy* image of the retinas stained represents a three-dimensional projection of the entire cryosection (sum of all images in the stack). For DJ-1 staining, sections were washed, slides were placed in a staining dish filled with Trilogy (Cell Marque, Rocklin, CA) and then the dish was placed into a steamer for 30 min., sections were then washed three times with PBS and blocked in PBS/BSA as previously described. Polyclonal antibodies to glucose transporter Glut-1 (Abcam, Cambridge, MA, diluted at 1∶200), Mn^+2^ superoxide dismutase (SOD2, Assay designs, Ann Arbor, MI, diluted at 1∶750) biliverdin reductase (Stressgen, Victoria, BC, Canada, diluted at 1∶500), PARK7 (Origene, Rockville, MD, diluted at 1∶750), monocarboxylate transporter 1 (MCT1, Millipore, Temecula, CA, diluted at 1∶1000), collectrin (Enzo Life Sciences, diluted at 1∶200) were used. Labeling for BVR and SOD2 was done according to the method described by Panahian and collaborators [38].

### Western Blot Analysis

Whole cell lysates from RPE collected from young (2 months) and old (24 months) rats were solubilized in RIPA buffer (0.1% SDS, 1% Triton X100, 1% deoxycholate, 0.15 M NaCl, 2 mM EDTA, 25 mM Tris pH 7.4) supplemented with a cocktail of protease and phosphatase inhibitors (Sigma Chemical Co., St. Louis, MO). Total RPE lysates (20 µg protein) were loaded into and resolved by SDS-PAGE on 4–20% Novex®-Tris-Glycine gel (Invitrogen Corporation, Carlsbad, CA) and electro-transferred to Immobilon PVDF membranes (Millipore, Bedford, MA). Membranes were blocked with HyBLOCKER liquid blocking reagent (Denville Scientific Inc., Metuchen, NJ) for 30 min. and incubated overnight in the same solution with antibodies to CD46 (sc-9098), DPPX (sc-46923), DDAH I (sc-26068), ezrin (sc-6409), all from Santa Cruz Biotechnology Inc. (Santa Cruz, CA); GABA 2 transporter (NB100-1872, Novus Biologicals, Littleton, CO); and cathepsin D (PAB12789, Abnova, Taipei, Taiwan). Protein detection was performed with secondary antibodies conjugated to peroxidase and visualized using ECL Plus Western Blotting detection reagent (GE Healthcare Bio-Sciences Corp, Piscataway, NJ). PVDF membranes were exposed to film, films were scanned and figures were composed using Adobe Photoshop CS3.

## Supporting Information

Figure S1 **Lectin labeling of eyecups from young and old F344BN RPE.** Both young (A, C) and old (B, D) eyecups with the exposed RPE were fixed and processed as wholemounts (A, B) or for cryosectioning (C, D). Whole mounts were labeled with WGA-FITC mounted on slides and observed in epifluorescence. 10 mm sections were labeled with WGA-FITC and nuclei were labeled with TO-PRO-3. RPE  =  retinal pigment epithelium; Ch  =  choroid. Bars  =  20 µm. Intensive lectin labeling is found in the apical surface of both samples.
(TIF)

Figure S2 **Age-related changes in RPE fractions networks.** Functional pathway and network analyses of RPE fractions were generated through the use of Ingenuity Pathway Analysis. Displayed are network analyses of protein interactions involved in lipid metabolism, molecular transport and small molecules biochemistry pathways in both young and old MV (A, B) and CB (C, D). A lower number of proteins was present in the aged CB fraction (). Proteins with the highest fold changes are shown in red while proteins with no fold changes are shown in white. Lines indicate protein-protein interactions. Dashed lines indicate protein expression. Orange lines and dashed lines connect different networks that were present with the same functions of the young MV fraction.
(TIF)

Table S1 List of proteins identified on young MV fraction of RPE cells.
(XLS)

Table S2 List of proteins identified on old MV fraction of RPE cells.
(XLS)

Table S3 List of proteins identified on young CB fraction of RPE cells.
(XLS)

Table S4 List of proteins identified on old CB fraction of RPE cells.
(XLS)

Table S5 Networks identified on MV fractions in both young and old F344BN rat RPE.
(DOC)

Table S6 Networks identified on CB fractions in both young and old F344BN rat RPE.
(DOC)
