# Supplementary figures and images for: Age-Related Changes in the Retinal Pigment Epithelium (RPE)
Source: PLoS One. 2012 Jun 11;7(6):e38673. doi: 10.1371/journal.pone.0038673 (PMC3372495; doi:10.1371/journal.pone.0038673)

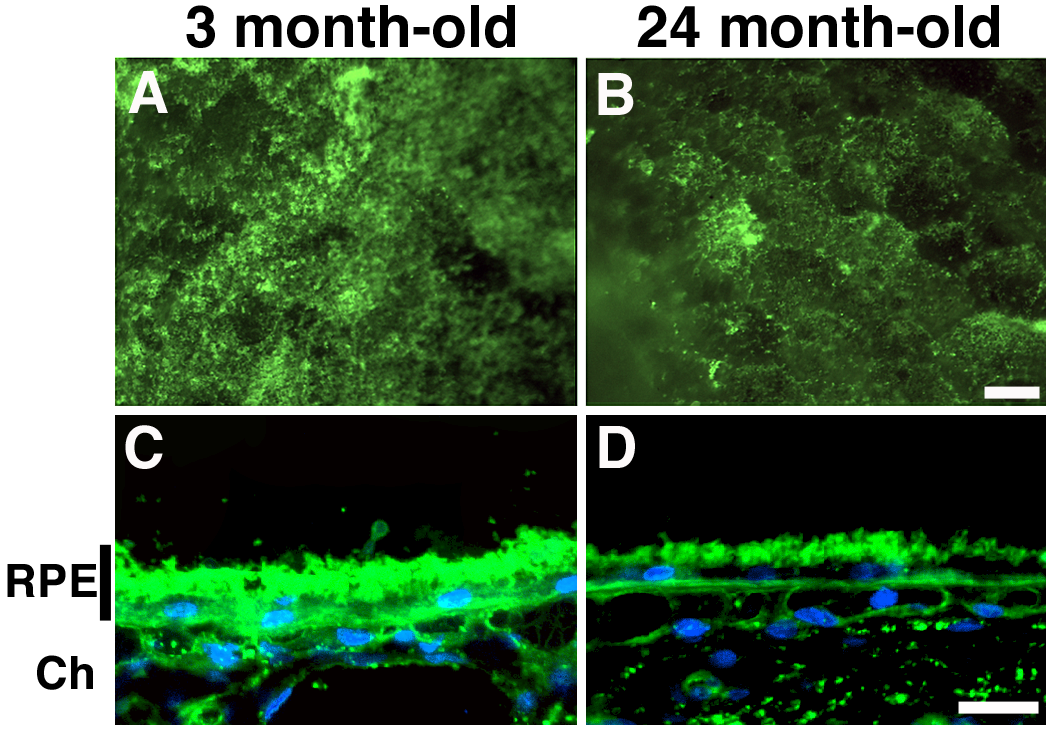

Supplement: Figure S1 — Lectin labeling of eyecups from young and old F344BN RPE. Both young (A, C) and old (B, D) eyecups with the exposed RPE were fixed and processed as wholemounts (A, B) or for cryosectioning (C, D). Whole mounts were labeled with WGA-FITC mounted on slides and observed in epifluorescence. 10 mm sections were labeled with WGA-FITC and nuclei were labeled with TO-PRO-3. RPE = retinal pigment epithelium; Ch = choroid. Bars = 20 µm. Intensive lectin labeling is found in the apical surface of both samples. (TIF) [file pone.0038673.s001.tif]

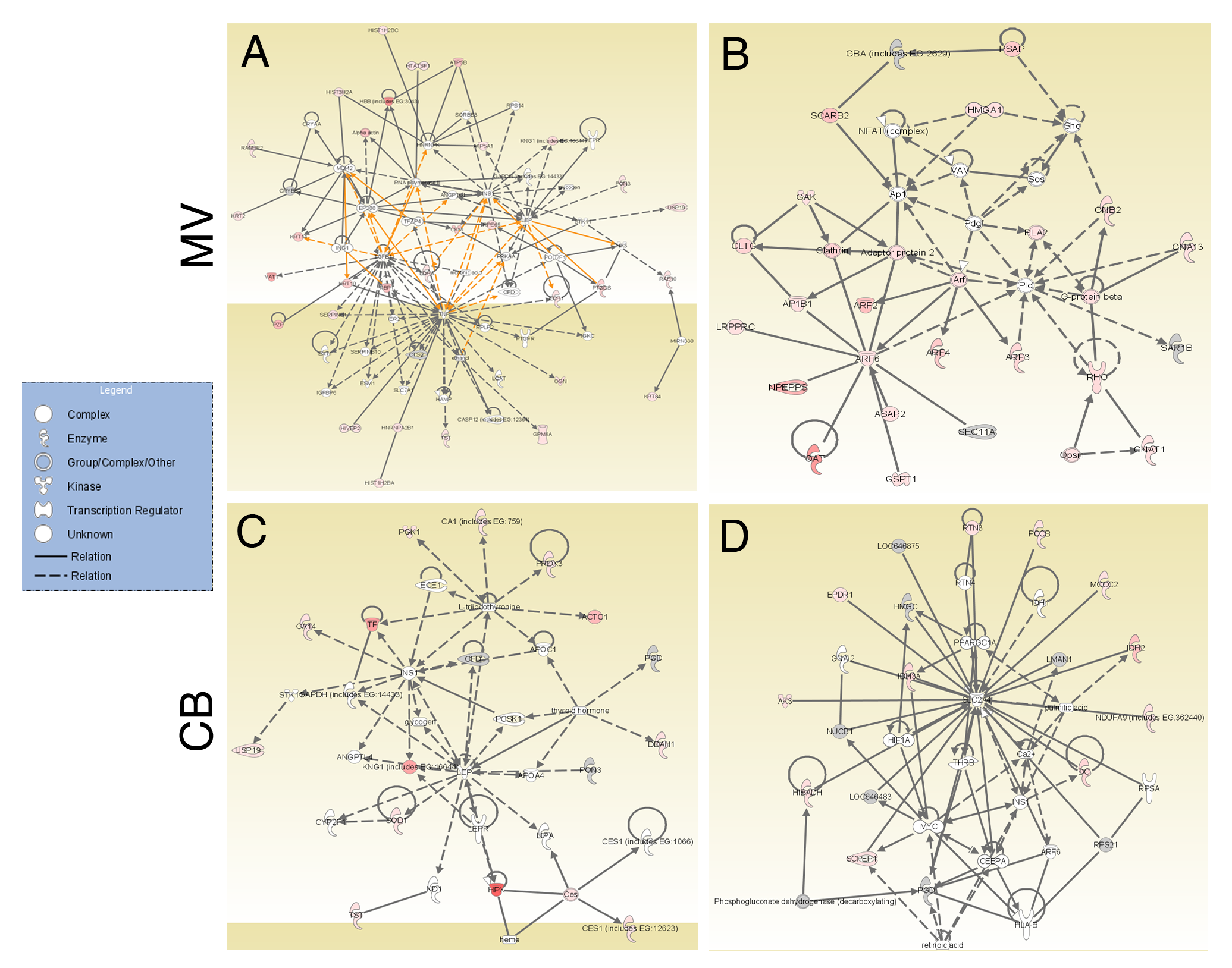

Supplement: Figure S2 — Age-related changes in RPE fractions networks. Functional pathway and network analyses of RPE fractions were generated through the use of Ingenuity Pathway Analysis. Displayed are network analyses of protein interactions involved in lipid metabolism, molecular transport and small molecules biochemistry pathways in both young and old MV (A, B) and CB (C, D). A lower number of proteins was present in the aged CB fraction (). Proteins with the highest fold changes are shown in red while proteins with no fold changes are shown in white. Lines indicate protein-protein interactions. Dashed lines indicate protein expression. Orange lines and dashed lines connect different networks that were present with the same functions of the young MV fraction. (TIF) [file pone.0038673.s002.tif]
