# Supplementary material for: Age-Related Changes in the Retinal Pigment Epithelium (RPE)
Source: PLoS One. 2012 Jun 11;7(6):e38673. doi: 10.1371/journal.pone.0038673 (PMC3372495; doi:10.1371/journal.pone.0038673)
Supplement: Table S5 — Networks identified on MV fractions in both young and old F344BN rat RPE. (DOC) [file pone.0038673.s007.doc]

**Supplementary Table 5.** Networks identified on MV fractions in both young and old F344BN rat RPE.

| **Fraction and Age^a^** | **Score** | **Focus Molecules** | **Network Pathways** |
| --- | --- | --- | --- |
|  | 45 | 28 | Small Molecule Biochemistry, Lipid Metabolism, Molecular Transport |
|  | 43 | 26 | Neurological Disease, Lipid Metabolism, Molecular Transport |
| **Young** | 41 | 25 | Cancer, Carbohydrate Metabolism, Cellular Movement |
|  | 40 | 26 | Cellular Assembly and Organization, Molecular Transport, Protein Trafficking |
|  | 39 | 25 | Genetic Disorder, Hematological Disease, Molecular Transport |
|  | 45 | 27 | Small Molecule Biochemistry, Free Radical Scavenging, Drug Metabolism |
|  | 44 | 28 | Hematological System Development and Function, Organismal Functions, Cancer |
| **Old** | 43 | 27 | Antigen Presentation, Carbohydrate Metabolism, Cardiovascular Disease |
|  | 42 | 27 | Genetic Disorder, Hematological Disease, Hematological System Development and Function |
|  | 42 | 26 | Cancer, Reproductive System Disease, Neurological Disease |
