# Supplementary material for: Age-Related Changes in the Retinal Pigment Epithelium (RPE)
Source: PLoS One. 2012 Jun 11;7(6):e38673. doi: 10.1371/journal.pone.0038673 (PMC3372495; doi:10.1371/journal.pone.0038673)
Supplement: Table S6 — Networks identified on CB fractions in both young and old F344BN rat RPE. (DOC) [file pone.0038673.s008.doc]

**Supplementary Table 6.** Networks identified on CB fractions in both young and old F344BN rat RPE.

| **Fraction and Age^a^** | **Score^b^** | **Focus Molecules^c^** | **Network Pathways^d^** |
| --- | --- | --- | --- |
|  | 47 | 33 | Genetic Disorder, Hematological Disease, Organismal Injury and Abnormalities |
|  | 47 | 33 | Protein Synthesis, Cancer, Cell Death |
| **Young** | 45 | 33 | RNA Post-Transcriptional Modification, Cellular Assembly and Organization, Cell-to-Cell Signaling and Interaction |
|  | 45 | 31 | Nucleic Acid Metabolism, Small Molecule Biochemistry, Energy Production |
|  | 43 | 30 | Molecular Transport, Protein Trafficking, Small Molecule Biochemistry |
|  | 48 | 32 | Cancer, Cell Death, Neurological Disease |
|  | 44 | 29 | Energy Production, Nucleic Acid Metabolism, Small Molecule Biochemistry |
| **Old** | 43 | 32 | Lipid Metabolism, Nucleic Acid Metabolism, Small Molecule Biochemistry |
|  | 40 | 29 | Auditory and Vestibular System Development and Function, Organ Development, Gene Expression |
|  | 39 | 27 | Lipid Metabolism, Nucleic Acid Metabolism,  Small Molecule Biochemistry |
